# Redox takes control

**DOI:** 10.7554/eLife.99765

**Published:** 2024-06-20

**Authors:** Iván Plaza-Menacho

**Affiliations:** 1 Kinases, Protein Phosphorylation and Cancer Group, Structural Biology Programme, Spanish National Cancer Research Centre (CNIO) Madrid Spain

**Keywords:** allostery, drug discovery, protein kinases, redox regulation, covalent inhibitors, conformation, *E. coli*, Human

## Abstract

A study of two enzymes in the brain reveals new insights into how redox reactions regulate the activity of protein kinases.

**Related research article** Bendzunas GN, Byrne DP, Shrestha S, Daly LA, Oswald SO, Katiyar S, Venkat A, Yeung W, Eyers CE, Eyers PA, Kannan N. 2024. Redox regulation of brain selective kinases BRSK1/2: Implications for dynamic control of the eukaryotic AMPK family through Cys-based mechanisms. *eLife* **13**:RP92536. doi: 10.7554/eLife.92536.

For decades reactive oxygen species were considered to be the by-products of cell damage. However, more recently these unstable molecules have been shown to play a role in cell signaling as secondary messengers that transfer electrons between proteins.

This exchange of electrons, known as a redox modification, typically takes place on a specific set of amino acids within the protein. Previous work showed that the removal of an electron (known as oxidation) from the amino acid cysteine can alter the structure and function of protein kinases (enzymes that phosphorylate molecules and have an essential role in cell signaling; Garrido Ruiz et al., 2022). As well as adding phosphoryl groups to other proteins, some kinases need to be phosphorylated themselves to switch on their catalytic activity (Cuesta-Hernández et al., 2023). However, much less is known about how cysteine oxidation modulates the activity and structure of protein kinases, and the systematic effect this has on cell signaling.

Now, in eLife, Natarajan Kannan and colleagues – including George Bendzunas and Dominic Byrne as joint first authors – report how cysteine oxidation controls two kinases found in the brain (Bendzunas et al., 2024). The team showed that the catalytic activity of these two enzymes – called AMPK-related Brain-selective kinases 1 (BRSK1) and 2 (BRSK2) – is directly regulated through reversible oxidation. This redox reaction takes place at cysteine residues situated at key structural and functional sites within the catalytic domain (the part of the protein that causes the enzymatic reaction).

In particular, Bendzunas et al. (who are based at the University of Georgia and the University of Liverpool) identified two pairs of cysteine residues in both BRSK1 and BRSK2, which form a bridge between sulfur atoms when oxidized. These connections, known as disulfide bonds, help maintain the structure and shape of proteins. When the team mutated the cysteine residues in vitro, this increased the catalytic activity of the kinases. It also led to higher amounts of phosphorylated Tau, the primary substrate of BRSK1 and BRSK2, in cells.

Molecular modelling and simulations revealed that oxidation of one of the four identified cysteines (which resides on a motif that defines the end of the activation loop) destabilizes bonds required to allosterically activate the catalytic domain. Taken together, these findings suggest that oxidation of the four cysteines, and subsequent formation of the two intramolecular disulfide bridges, represses the activity of BRSK1 and BRSK2.

Many other protein kinases have cysteine residues situated in similar locations within their structure. It is therefore possible that redox regulation of disulfide bridges may be a widespread mechanism for controlling protein kinase activity and signaling.

In a previous study led by Kannan, roughly 10% of protein kinases present in humans were hypothesized to be subjected to redox-dependent regulation (Byrne et al., 2020). This includes key members of the CAMK, AGC, and AGC-like families, which each contain a cysteine residue that lies adjacent to the phosphorylation site in the activation loop. The transmembrane receptor EGFR also has a cysteine residue in another nearby location, which enhances catalytic activity when oxidized (Truong et al., 2016). However, it is unclear how the redox state of these cysteine residues modulates the activity of kinases. As well as altering the conformational landscape of the activation loop itself, redox modifications may enhance the non-catalytic properties of the enzyme, or regulate intermolecular interactions and oligomerization (Cuesta-Hernández et al., 2023).

Cysteine residues have also been found in other parts of the kinase structure. Comprehensive mapping revealed a group of cysteine residues that lie in or adjacent to the binding site for ATP, which are collectively referred to as the ‘cysteinome’ (Chaikuad et al., 2018). Furthermore, the protein kinases cAPK, c-Src and LRRK2 among others, have all been shown to contain two cysteine residues which alter catalytic activity following redox modifications (Humphries et al., 2005; Humphries et al., 2002; Heppner et al., 2018; Trilling et al., 2024). Many of the motifs that contain cysteine residues also have a phosphorylation site, but it is poorly understood how phosphorylation impacts the oxidation of cysteines (Kemper et al., 2022).

These redox-dependent molecular switches provide therapeutic advantages as they can be used to block the activity of kinases. Drugs targeting cysteine residues, known as covalent inhibitors, were initially designed for members of the EGFR family (Tsou et al., 2005). Since then, more than forty covalent inhibitors have been approved by the US Food and Drug Administration (FDA). Most of these target residues in the cysteinome, particularly a cysteine at the front site of the kinase in the F2 position (Chaikuad et al., 2018). However, other cysteine hotspots are yet to be fully explored (Yen-Pon et al., 2018; Chen et al., 2022; Zhang et al., 2016), including the cysteine disulfide bonds identified by Bendzunas et al. which could be important therapeutic targets.

The work of Bendzunas et al. and others highlights how significant redox biology is for understanding protein kinase function and pharmacology. However, the regulatory power of cysteine oxidation is often underappreciated, and the effect it has on most human kinases remains unknown. Further exploration of the cysteines in protein kinases, together with more high-resolution structural data, will help to bridge this gap and may lead to the discovery of more drug targets for covalent inhibitors.
